# Effect of Coronary Slow Flow on Intrinsicoid Deflection of QRS Complex

**DOI:** 10.1155/2018/2451581

**Published:** 2018-02-01

**Authors:** Sabri Seyis

**Affiliations:** Department of Cardiology, Liv Hospital, Istinye University, Istanbul, Turkey

## Abstract

Coronary slow flow is a rare, clinically important entity observed in acute coronary syndrome. The pathophysiological mechanism is not fully elucidated. We investigated patients with chest pain who had angiographic features consistent with the coronary slow flow. One hundred ten patients were included. Electrocardiography, echocardiography, and angiography results were retrospectively noted. The mean age was 56.4. Fifty-eight were male, and fifty-two were female. The control group consisted of patients with normal angiography. Patients had higher diastolic blood pressure, lower mean ejection fraction, higher average left ventricular end-diastolic diameter, and higher mean left atrial size than the control group (*p*=0.009, *p*=0.017, *p*=0.041, and*p* < 0.001, resp.). Patients had higher average V1 ID, V6 ID, P wave dispersion, TFC LAD, TFC Cx, TFC RCA, and TFC levels than the control group. A significant linear positive relationship was found between the V1 ID and the TFC LAD, TFC Cx, TFC RCA, and TFC; also between the V6 ID and the TFC LAD, TFC Cx, TFC RCA, and TFC. Angiographic and electrocardiographic features are suggestive and diagnostic for the coronary slow flow syndrome. Although when regarded as a benign condition, coronary slow flow should be diagnosed, followed up, and treated as many of laboratory features suggest ischemic events.

## 1. Introduction

Coronary slow flow (CSF) is an important clinical entity which may cause precordial pain during rest or exercise, and it is defined as a microvascular disorder characterized by the slow progression of the administered opaque material to the distal vasculature during coronary angiography without the obstruction of the epicardial coronary arteries [1]. It has been reported that CSF is observed in 1% of patients who underwent coronary angiography with a diagnosis of the acute coronary syndrome (ACS) [2]. The underlying mechanism of CSF, its pathophysiologic mechanism, and its clinical significance have not been fully elucidated [1]. Tambe et al. who first described CSF in 1972 suggested that this phenomenon might be due to anomalies in the coronary microcirculation [3]. It is clinically different from other coronary microvascular diseases [4]. Microvascular dysfunction, endothelial and vasomotor dysfunction, occlusive diseases of small coronary arteries, and myocardial ischemia are associated with the impaired coronary blood flow, and they are proposed mechanisms in CSF [5–8].

As demonstrated by all these pathogenetic reasons, it is inevitable that patients with CSF have ischemia and angina pectoris as its clinical manifestation. The presence of ischemia has been confirmed in 30–80% of these patients by the metabolic processes such as myocardial lactate formation and O_2_ use and by thallium-201 myocardial perfusion scan (MPS) with exercise electrocardiography (ECG) [8–16]. Although patients with normal coronary arteries and chest pain are generally thought to have a good prognosis, many studies have shown that these normal looking arteries are not innocent since the symptoms continue and patients may be candidates for acute coronary syndrome [13, 17, 18].

The time from the beginning of the QRS complex to the R waves or the peak of the R′ waves in a 12-lead ECG is called R-wave peak time (RWPT) or intrinsicoid deflection (ID) time. This parameter, in fact, reflects the spread of the electrical activity in the heart from the endocardium to the epicardium. It is a simple ECG measurement that can be used to facilitate diagnosis and determine prognosis in many different cardiac diseases [19]. It has been shown that ID increases especially in diastolic dysfunction situations where diastolic wall tension increases. Tissue Doppler imaging (TDI) studies have also shown that there is a significant relationship between ID and diastolic dysfunction parameters.

Although there are many studies suggesting that the CSFP disrupts the systolic and diastolic functions, there are also publications claiming the opposite. In this study, we investigated ID, a simple indicator of diastolic dysfunction and electrical activation impairment in particular, in patients with CSF.

## 2. Methods

### 2.1. Patient Selection

Patients who were admitted to our clinic with complaints of chest pain and underwent coronary angiography with appropriate indications are included. One hundred ten patients who had slow coronary flow during angiography were included in the study. Angiography images and ECG were examined retrospectively. The patients' demographics and echocardiographic findings were recorded. And the patients who were found to have normal coronary arteries during angiography were selected for the control group. Patients with permanent pacemaker implantation, right and left bundle branch block, atrial fibrillation history, advanced stage pulmonary disease, end-stage heart failure, heart valve disease, and using antiarrhythmic medications were excluded.

### 2.2. Echocardiographic Evaluation

Echocardiographic evaluation was performed using a 2-dimensional M-mode cardiovascular ultrasound system (GE-Vingmed Vivid 7 System, Germany). The examination was performed in the standard left lateral decubitus position (LLDP). Parasternal long axis, short axis, and apical four-cavity views were used for measurements. The ejection fraction was calculated using the modified Simpson method. All echocardiography procedures were performed by an experienced cardiologist, and the findings were recorded.

### 2.3. Electrocardiography

ECGs (Cardiovit AT-102 Plus, Schiller, Switzerland) were recorded at a standard rate of 25 mm/s and an amplitude of 10 mm/mV. These were routine ECGs recorded before the patients underwent the angiography procedure. All ECGs were scanned at 800 dpi resolution and transferred to the computer. The ID measurements in V1 and V6 were performed on the computer by an experienced cardiologist, and the measurement results were averaged. The time from the beginning of the earliest Q or R wave to the peak of the R wave was defined as ID.

P wave duration was measured for all leads. The beginning of the P wave was the first upward or downward departure from the baseline for the positive and negative waveforms, respectively. The return to the baseline was accepted at the end of the P wave. The difference between P_max_ and P_min_ was defined as P wave dispersion.

### 2.4. Coronary Angiography and Documentation of the TIMI Frame Count

For all the participants in this study, selective coronary angiography was performed using standard Judkins technique. Coronary arteries were imaged at right and left oblique positions using cranial and caudal angles at a rate of 25 fps by manually injecting an average of 6–8 ml of opaque material for each take. Coronary flow was quantified objectively using the TIMI (thrombolysis in myocardial infarction) frame count (TFC) method as described by Gibson et al. [20, 21]. For the TFC measurement, the moment when the contrast agent touched both sides of the coronary artery and begun to move forward was chosen as the starting point; and as the endpoint, the moment when the contrast agent reached the so-called moustache point was chosen for the left anterior descending (LAD); the moment when the first side branch arises in posterior lateral artery was chosen for the right coronary artery (RCA); and the moment when distal bifurcation of the longest branch was displayed was chosen for the left circumflex artery (Cx). Since LAD lasts longer, the measured value was standardized via dividing by 1.7 [21, 22]. The mean of TFC for each participant was calculated by dividing the sum of the TFCs of LAD (corrected), LCX, and RCA by 3. All participants with a corrected TFC greater than 27 (two standard deviations from the published normal range) in at least one of the three epicardial coronary arteries were accepted as having CSF, while those whose TFC fell within the standard deviation of published normal range were considered as having normal coronary flow [21].

### 2.5. Statistical Analysis

From the summary of data, descriptive statistics were tabulated as mean ± standard deviation for continuous variables. Categorical variables were summarized as number and percentage. The *Shapiro–Wilk test* was used in testing the normality distribution of the numerical variables when *n* < 50, the *Kolmogorov–Smirnov test* was used when *n* > 50, and *the independent samples t-test*, a parametric test, was used in comparisons of two independent groups when there was a normal distribution. The *Pearson chi-square test* was used in comparisons of differences between categorical variables. *Pearson correlation coefficient* was used in the evaluation of relationships between numerical variables when there was a normal distribution. Statistical analyses were performed with the R 3,3,2v Program (open source), and the level of significance in the statistical analyses was *p* < 0.05.

## 3. Results

The demographics of the patient and control groups are summarized in Table 1. The patient and control groups were not significantly different in terms of the following characteristics: age, gender distribution, BMI, smoking, aspirin use, renin-angiotensin system (RAS) blocker use, beta-blocker use, statin use, heart rate, systolic blood pressure, left ventricular end-systolic diameter (LVESD), interventricular septal diameter (IVSD), and left ventricle posterior wall thickness (LVPWT) (*p* > 0.05).

Diastolic blood pressure was higher in the patient group (75 ± 6.6) than the control group (72.8 ± 5.67) (*p*=0.009). The mean ejection fraction (EF) of the control group (62.44 ± 3.08) was found significantly higher than that of the patient group (*p*=0.017). The average left ventricular end-diastolic diameter (LVEDD) of the patient group (49.61 ± 3.17) was higher than that of the control group (48.78 ± 2.78) (*p*=0.041). The mean left atrial (LA) size of the patient group (36.87 ± 1.52) was higher than that of the control group (36.03 ± 1.84) (*p* < 0.001). The average V1 ID of the patient group (45.85 ± 5.72) was significantly higher than that of the control group (27.44 ± 4.38). The average V6 ID of the patient group (65.61 ± 5.86) was significantly higher than that of the control group (38.58 ± 28.89). The average P-wave dispersion (PWD) of the patient group (43.18 ± 7.54) was higher than that of the control group (20.71 ± 3.94). The average TFC LAD of the patient group (42.24 ± 8.6) was higher than that of the control group (16.75 ± 1.16). The average TFC Cx of the patient group (34.97 ± 5.16) was found significantly higher than that of the control group (15.01 ± 0.8). The average TFC RCA of the patient group (31.03 ± 2.86) was higher than that of the control group (14.17 ± 0.81). The average TFC of the patient group (36.04 ± 5.31) was higher than that of the control group (15.28 ± 0.61).

A significant linear positive relationship was found (*p* < 0.05) between the V1 ID and the TFC LAD, TFC Cx, TFC RCA, and TFC, as shown in Table 2. It appears that V1 ID increases as TFC LAD, TFC Cx, TFC RCA, and TFC increase. Likewise, there was a significant linear positive relationship between the V6 ID and the TFC LAD, TFC Cx, TFC RCA, and TFC (*p* < 0.05). The V6 ID was found to increase with increases in the TFC LAD, TFC Cx, TFC RCA, and TFC.

## 4. Discussion

Coronary slow flow phenomenon (CSFP) is not a rare condition. CSF was detected in 7% of the patients who came to clinic with chest pain and underwent angiography due to a suspicion of coronary artery disease [23]. The clinical significance and the underlying pathophysiological mechanism of CSF have not been fully elucidated. The occlusion of small vessels, increased microvascular resistance, and atherosclerosis are suggested mechanisms. Recently, significant findings have been published indicating a role for endothelial activation and inflammation [24]. In a study involving endomyocardial biopsy of the left ventricle, vessel wall thickening leading to luminal narrowing, mitochondrial abnormalities, and a decrease in glycogen content were found [25]. In addition, extensive calcification with no luminal narrowing, diffuse intimal thickening, and atheromatous plaques on the vascular wall were found at the full length of epicardial coronary arteries in studies of the patients with CSF [26–28]. In another study, flow-mediated dilatation in the brachial artery was found to be impaired in patients with CSF [7]. High concentrations of endothelin 1 and low concentrations of nitric oxide were found in peripheral blood and coronary sinus of the patients with CSF after resting and stress; thus, their endothelial functions were shown to be impaired [9, 29, 30]. In light of these findings, it is not off the mark to say that CSF is associated with ischemia. Studies with myocardial perfusion scan and cardiac stress test have already shown ischemia in 30–80% of these patients [8–16]. There are publications showing that it may lead to stable angina pectoris and myocardial infarction, and even malignant arrhythmias and sudden deaths. In a study by Sezgin et al., diastolic dysfunction was found in patients with CSF [31].

In a study by Elsherbiny, impairment in both systolic and diastolic functions was found in patients with CSF [32]. In a study by Baykan et al., systolic and diastolic dysfunction was shown in patients with CSF in the evaluations performed with tissue Doppler imaging (TDI), and it was suggested to evaluate them with TDI instead of standard echocardiography [33]. The reason for our inability to detect diastolic dysfunction in the patients with CSF in our study might be the use of echocardiography instead of TDI.

ID measurement is a very simple ECG indicator for detection of diastolic dysfunction. In the study by Boles et al., ID prolongation was detected in the early stages of diastolic dysfunction [34]. It has been proven that the diastolic function was impaired initially in the presence of ischemia [35]. Indeed, it may be said that the changes in electrical activity had begun before the manifestation echocardiographic findings. In the study by O'neal et al. that followed up patients over the course of 11 years, it was shown that ID prolongation in people without heart disease is a predictor of heart failure that may develop in the future and that it is more powerful than the QRS duration in predicting the prognosis [36]. Every 10 ms prolongation increases the risk 1.4 fold. Darouian et al. found that there was a relationship between the prolonged ID and sudden cardiac death and that this association was independent of the left ventricular mass [37].

In our study, the ID of the patients with CSF was significantly prolonged. The average ID measurement was longer in both the V1 and V6 leads of the patient group. Interestingly, the ID measurement in V6 was correlated with the slow flow in LAD and Cx arteries, and the ID measurement in V1 was correlated with the slow flow in RCA. This result may be due to the fact that V1 represents mainly the right ventricle, and the right ventricle is often nourished by RCA.

In addition, we found that the more the degree of slow flow, or in other words the higher the TFC is present, the higher the ID is found. This suggests that the risk of developing heart failure in the future is higher as the degree of CSF is higher in patients.

It has been shown that autonomic cardiac function is impaired and sympathetic activity is increased in coronary artery disease [38]. Some authors view CSF as a subgroup of syndrome X. From this point of view, impaired autonomic functions may be thought to prolong the duration of ventricular activation.

## 5. Conclusions

Conclusively, CSF actually contains subclinical findings as shown in our study although it was thought to be a benign phenomenon by many clinicians. In addition to being a practical method for early detection, the prognostic importance of ID prolongation is recently being more emphasized. Therefore, patients with ID prolongation and CSF should be followed closely and further follow-up is needed in terms of treatment.

## Figures and Tables

**Table 1:** The demographics of the patient and control groups.

	Group		
	Control	Patient	*p*
*Age* ^1^	56.69 ± 6.73	56.35 ± 7.51	0.730
*Sex* ^2^			
Female	47 (43.12)	52 (47.27)	0.537
Male	62 (56.88)	58 (52.73)	
*BMI* ^1^	26.6 ± 2.4	26.55 ± 2.87	0.887
*Smoking* ^2^			
No	78 (71.56)	72 (65.45)	0.331
Yes	31 (28.44)	38 (34.55)	
*Aspirin* ^2^			
No	75 (68.81)	73 (66.36)	0.699
Yes	34 (31.19)	37 (33.64)	
*RAS blocker* ^2^			
No	56 (51.38)	57 (51.82)	0.948
Yes	53 (48.62)	53 (48.18)	
*Beta blocker* ^2^			
No	68 (62.39)	69 (62.73)	0.958
Yes	41 (37.61)	41 (37.27)	
*Statin* ^2^			
No	66 (60.55)	70 (63.64)	0.638
Yes	43 (39.45)	40 (36.36)	
*KKB* ^2^			
No	84 (77.06)	85 (77.27)	0.971
Yes	25 (22.94)	25 (22.73)	
Heart rate^1^	66.96 ± 6.71	67.35 ± 7.21	0.685
Systolic BP^1^	123.17 ± 6.03	124 ± 6.2	0.314
Diastolic BP^1^	72.8 ± 5.67	75 ± 6.6	**0.009**
EF^1^	62.44 ± 3.08	61.41 ± 3.28	**0.017**
LVEDD^1^	48.78 ± 2.78	49.61 ± 3.17	**0.041**
LVESD^1^	34.89 ± 2.84	35.5 ± 3.49	0.158
IVSD^1^	10.64 ± 0.86	10.62 ± 0.94	0.843
LVPWT^1^	9.69 ± 0.6	9.78 ± 0.75	0.308
LA^1^	36.03 ± 1.84	36.87 ± 1.52	**<0.001**
ID V1^1^	27.44 ± 4.38	45.85 ± 5.72	**<0.001**
ID V6^1^	38.58 ± 28.89	65.61 ± 5.86	**<0.001**
PWD^1^	20.71 ± 3.94	43.18 ± 7.54	**<0.001**
TFC LAD^1^	16.75 ± 1.16	42.24 ± 8.6	**<0.001**
TFC Cx^1^	15.01 ± 0.8	34.97 ± 5.16	**<0.001**
TFC RCA^1^	14.17 ± 0.81	31.03 ± 2.86	**<0.001**
TFC^1^	15.28 ± 0.61	36.04 ± 5.31	**<0.001**

^1^Independent sample *t-*test; ^2^chi-square test.

**Table 2:** Relationships between the parameters.

	*N*	*r*	*p*
ID V1 & TFC LAD	110	0.875	<0.001
ID V1 & TFC CX	110	0.894	<0.001
ID V1 & TFC RCA	110	0.838	<0.001
ID V1 & TFC	110	0.912	<0.001
ID V6 & TFC LAD	110	0.911	<0.001
ID V6 & TFC CX	110	0.933	<0.001
ID V6 & TFC RCA	110	0.802	<0.001
ID V6 & TFC	110	0.938	<0.001

Pearson correlation coefficient was used.
